# Silencing of microRNA-101 prevents IL-1β-induced extracellular matrix degradation in chondrocytes

**DOI:** 10.1186/ar4114

**Published:** 2012-12-10

**Authors:** Linghui Dai, Xin Zhang, Xiaoqing Hu, Chunyan Zhou, Yingfang Ao

**Affiliations:** 1 Institute of Sports Medicine, Peking University Third Hospital, 49 North Garden Road, Haidian District, Beijing 100191, P. R. China; 2 Department of Biochemistry and Molecular Biology, Peking University School of Basic Medical Sciences, 38 Xueyuan Road, Haidian District, Beijing 100191, P. R. China

## Abstract

**Introduction:**

Extracellular matrix (ECM) degradation leads to malfunction of the cartilage in osteoarthritis (OA). Inflammatory cytokine interleukin-1 beta (IL-1β) functions in ECM degradation and prevents ECM synthesis by down-regulating the key transcription factor, *Sox9*, and consequently inhibiting ECM gene expression. Evidence reveals that microRNAs (miRNA) have been associated with OA, but little is known of their function in chondrocyte ECM degradation. This study aimed to identify possible miRNAs that mediate IL-1β-induced down-regulation of *Sox9 *as well as its known down-stream genes, *collagen type II *and *aggrecan*.

**Methods:**

The miRNAs were predicted based on three classical databases. The expression levels of the predicted miRNAs were assessed in IL-1β stimulated chondrocytes by real-time PCR. A luciferase reporter was used to test the binding of the miRNAs to the 3' untranslated regions (3'UTR) of *Sox9*. The predicted miRNAs were transfected into chondrocytes to validate their relationship with *Sox9*. Functional analysis of the miRNAs on chondrocytes ECM degradation was performed at both the mRNA and protein levels after miRNA transfection and IL-1β treatment.

**Results:**

Six miRNAs were predicted to target *Sox9*, and their expression in IL-1β-stimulated chondrocytes was revealed by real-time PCR. The luciferase reporter assay indicated that only miR-101 could bind to the 3'UTR of *Sox9*. The expression of *Sox9 *was likewise negatively regulated by miR-101 in rat chondrocytes. Functional analysis showed that miR-101 could aggravate chondrocyte ECM degradation, whereas miR-101 inhibition could reverse IL-1β-induced ECM degradation.

**Conclusion:**

miR-101 participates in IL-1β-induced chondrocyte ECM degradation. Down-regulating miR-101 expression can prevent the IL-1β-induced ECM degradation in chondrocytes. miR-101 probably functions by directly targeting *Sox9 *mRNA.

## Introduction

Articular cartilage is composed of a small number of chondrocytes and a large amount of extracellular matrix (ECM). Chondrocytes are the only cell types in cartilage that function in the synthesis and catabolism of the ECM. The ECM, which mainly consists of collagen type II and aggrecan, maintains the structure of the cartilage as well as the homeostasis in its extracellular environment [1]. During osteoarthritis (OA), the degeneration and insufficient synthesis of ECM cause the cartilage to malfunction [1,2]. The inflammatory cytokine IL-1β has a key function in the cartilage degradation during OA [3]. IL-1β stimulates the synthesis of ECM-degrading enzymes, such as collagenases and aggrecanase, thereby leading to breakdown of the chondrocyte ECM [4-6]. On the other hand, IL-1β strongly inhibits the expression of cartilage-specific genes, such as *collagen type II *and *aggrecan*, and causes the insufficient synthesis of chondrocyte ECM [7,8]. In this process, cartilage-specific gene expression is inhibited via the down-regulation of *Sox9*, a transcription factor that can directly promote the expression of *collagen type II *and *aggrecan *[9-11]. The decreased *Sox9 *expression can lead to down-regulation of *collagen type II *and *aggrecan *in the presence of inflammatory cytokines such as IL-1β [7] and IL-6 [12]. The poor healing capacity of cartilage can be caused by inhibited *Sox9 *expression [7]. Therapeutic strategies aim to develop biological agents that block these two processes, thereby protecting chondrocytes from inflammatory cytokine-induced ECM degradation.

miRNAs have attracted attention because of their crucial roles in human disease and their potential as therapeutic targets [13-15]. miRNAs are small noncoding RNAs that can silence target mRNAs by binding to complementary sequences in 3' untranslated regions (3'UTR) to induce target mRNA degradation or translational repression [16]. miRNAs have been associated with the collagenases and aggrecanase that are stimulated by IL-1β in OA cartilage degradation [17-19]. However, little is known about the functions of miRNAs in IL-1β-induced down-regulation of *collagen type II *and *aggrecan *genes in cartilage. Understanding these processes will provide new insights into a therapeutic strategy to prevent cartilage damage.

We hypothesize that some miRNAs can participate in chondrocyte ECM degradation by regulating *Sox9 *expression in the presence of IL-1β. In this study, we selected six miRNAs from public miRNA databases; these miRNAs were predicted to target the *Sox9 *gene and demonstrated the direct targeting of *Sox9 *mRNA by miR-101. The functional analysis demonstrated that miR-101 could aggravate chondrocyte ECM degradation. The inhibition of miR-101 increased the expression of *Sox9, collagen type II *and *aggrecan*, and could also prevent chondrocyte from IL-1β-induced ECM degradation.

## Materials and methods

### Isolation of rat chondrocytes

Chondrocytes were isolated from the femoral condyle and tibial plateau of Sprague-Dawley rats (150 g to 160 g). All rats were obtained from Beijing Animal Administration Center. Ethical approval was obtained from the Animal Care and Use Committee of Peking University (number LA2010-065). Rat articular cartilage was cut into small fragments, followed by digestion first with 0.25% trypsin (Invitrogen, Carlsbad, CA, USA) for 30 minutes and then with 0.3% collagenase type II (Invitrogen) for 4 h at 37°C. Then cells were suspended in DMEM (Invitrogen) with 10% fetal bovine serum (HyClone Laboratories, Losan, UT, USA), 100 units/ml penicillin, and 100 units/ml streptomycin. Chondrocytes were cultured at 37°C in a humidified atmosphere of 5% carbon dioxide and 95% air. Primary chondrocytes at 80% confluence were used for all the studies described here.

### miRNA transfection and IL-Iβ stimulation

A total of 2 × 10^5 ^chondrocytes in 2 ml DMEM were incubated to 80% confluence in a 6-well plate and then changed to serum-free DMEM for 12 h incubation. The transfection of miRNA was performed according to the manufacturer's instruction. Briefly, 100 nM miRNA mimic or 100 nM scrambled 22 nt nucleotides (miR-Scr, with no homology to mammal genome) or 150 nM inhibitors (designed and synthesized by RiboBio, Guangzhou, China) were mixed with Lipofectamine 2000 (Invitrogen) and then left at room temperature for 20 minutes. Before the mixture was added, 1 ml fresh medium was added to each well, and then the mixture was added for 12 h incubation; 5 ng/ml IL-1β (PeproTech, Rocky Hill, NJ, USA) or PBS was added to each well and incubated for an appropriate period.

### RNA isolation and real-time PCR analysis

Total RNA was extracted using TRIzol reagent (Invitrogen). Isolated RNA was reverse-transcribed with a commercial kit (Promega, Madison, WI, USA), and real-time PCR analysis was performed using the Mx3005 QPCR System (Agilent Technology, Palo Alto, CA, USA) with SYBR Green PCR Master Mix (Toyobo, Osaka, Japan). The conditions of real-time PCR were as follows: 95°C for 2 minutes, followed by 40 cycles of 95°C for 15 sec and 60°C for 30 sec. A dissociation stage was added at the end of the amplification procedure. There was no nonspecific amplification determined by the dissolved curve. The PCR primers were as follows: *Sox9 *forward (FW), 5'-AGGAAGCTGGCAGACCAGTA-3' and reverse (RV), 5'- ACGAAGGGTCTCTTCTCGCT-3'; *Collagen type II *FW, 5'-CACCGCTAACGTCCAGATGAC-3', and RV, 5'-GGAAGGCGTGAGGTCTTCTGT-3'; *Aggrecan *FW, 5'-CCACTGGAGAGGACTGCGTAG-3' and RV, 5'- GGTCTGTGCAAGTGATTCGAG-3'; 18s RNA FW, '-GTAACCCGTTGAACCCCATT-3', and RV, 5'-CCATCCAATCGGTAGTAGCG-3'.

For analysis of miR-101 expression, reverse transcription and PCR were carried out using Bulge-Loop™ miRNA qPCR Primer Set (RiboBio) according to the manufacturer's instructions. The expression of *Sox9, Collagen type II*, and *Aggrecan *relative to 18s RNA and the miRNA expression relative to U6 (RiboBio) were determined using the 2^-ΔΔ^CT method [20].

### Protein isolation and western blotting

Protein was extracted using lysis buffer (50 mM Tris-HCl, pH 7.4, 150 mM NaCl, 1% NP- 40, and 0.1% sodium dodecyl sulfate), and the concentration was measured using the BCA protein assay kit (Pierce, Rockford, IL, USA) using bovine serum albumin as the standard. Proteins were run on SDS-PAGE gels (10%) and electro-transferred to nitrocellulose membrane at 4°C for 2 h. The blots were probed with anti-*Sox9 *(Millipore, Temecula, CA, USA) at 1:4000 dilutions overnight at 4°C, followed by incubation with horseradish peroxidase-conjugated secondary antibody (Santa Cruz, Santa Cruz, CA, USA, 1: 1000 dilutions) at room temperature for 1 h. Proteins were detected by chemiluminescence according to the manufacturer's recommendations (ECL, Millipore). Glyceraldehyde-3-phosphate dehydrogenase (GAPDH) was used as an internal control.

### Luciferase reporter construction, transfection, and dual luciferase assay

The 3' UTR of rat *Sox9 *gene [XM_001081628: GenBank] was PCR-amplified from rat genomic DNA using primers 5'-CCGCTCGAGGGAGACCTTGAAGAGCAATGG-3' and 5'-GAATGCGGCCGCCTTTCTCTCTTTCTCTCTTTCTTTTTTTAAGG-3', and cloned into the Xhol and Notl sites of pmiR-RB-REPORT (Promega), which was designated pmiR-*Sox9*-wt after sequencing. Site-directed mutagenesis of the miR-101 target-site in the *Sox9 *3'UTR was carried out using a site-directed mutagenesis kit (Takara Shuzo, Kyoto, Japan), with pmiR-Sox9-wt as a template. It was named pmiR-*Sox9*-mt (primers: FW, 5'-CTTTTAGTATGTACTACGTATGACTCA-3', RV, 5'-GTAGTACATACTAAAAGTATTTAAAAT-3').

HeLa cells were transfected with 300 ng of UTR reporter (pMir-Report, Promega), 10 ng of control Renilla vector (phRLTK, Promega), and 50 nM microRNA mimic with 1.5 μl Lipofectamine 2000 in each well of the 24-well plates. Lysates were harvested 24 h after transfection, and reporter activity was measured with Dual Luciferase Assay (Promega).

### Sulfated-glycosaminoglycan quantification

Cell suspension was analyzed for soluble sulfated-glycosaminoglycan (sGAG) secretion/formation by dimethylmethylene blue (DMMB) assay according to an established protocol [21-23]; Briefly, 20 μl of cell suspension was mixed with 200 μl of DMMB reagent, and the absorbance was measured at the 525 nm wavelength on the FlexStation III (Molecular Devices, Sunnyvale, CA, USA). A standard curve based on chondroitin 6-sulfate from shark (Sigma, St. Louis, MO, USA) was established to compare absorbance of the samples. Total sGAG were normalized to total protein content in the cell lysate of each group that was measured using the BCA protein assay kit (Pierce).

### Immunofluorescence analysis

Cultured cells were rinsed in PBS and fixed with 3.7% formaldehyde in PBS for 10 minutes at room temperature. Goat serum was used to block nonspecific binding sites. The cultured cells were then incubated with anti-*Collagen type II *(Abcam, Cambridge, UK, 1:100 dilutions) in PBS for 2 h at room temperature. After three PBS washes, each for 5 minutes at room temperature, the cells were incubated for 30 minutes with goat anti-rabbit IgG conjugated to fluorescent cy5 dye (Abcam, 1:100 dilutions) in PBS. After another round of three washes, the samples were incubated with Hoechst 33342 for 5 minutes. After the final round of three washes, samples were mounted and observed under a confocal microscope (FV 1000 Olympus IX-81, Olympus, Tokyo, Japan). Images were analyzed using Image-Pro Plus 6.0 software (Media Cybernetics, Silver Spring, MD, USA).

### Construction of *Sox9 *plasmids and *Sox9 *knockdown by small interfering RNA

*Sox9 *full-length vector and *Sox9 *CDS vector were purchased from SinoGeneMax (Beijing, China). Small interfering RNA (siRNA) against *Sox9 *(siSox9) and the scrambled siRNA (siScr) were designed and synthesized by RiboBio. The transfection of *Sox9 *full-length, *Sox9 *CDS vector, siScr and siSox9 in chondrocytes was carried out using Lipofectamine 2000 (Invitrogen) and performed according to the manufacturer's protocol.

### Northern blot analysis

The northern blot analysis was performed with miRNA Northern Blot Assay Kit (Signosis, Inc., Sunnyvale, CA) following the manufacturer's instructions. The oligonucleotide probes used to detect miR-101 and U6 snRNA are: miR-101, 5'- TTCAGTTATCACAGTACTGTA and U6, 5'-AACGCTTCACGAATTTGCGT, as previously reported [24]. U6 was used as an internal control.

### Statistical analysis

In each experiment the samples were analyzed in triplicate. Three independent experiments were performed, each with different chondrocyte preparation. The statistical significance of the differences between groups were calculated using analysis of variance (ANOVA). The results from the same group were evaluated using Student's *t*-test. *P*-values less than 0.05 were considered statistically significant. All data are presented as mean ± SD.

## Results

### miRNA prediction and expression of miRNA in IL-1β treated chondrocytes

**Figure 1:**
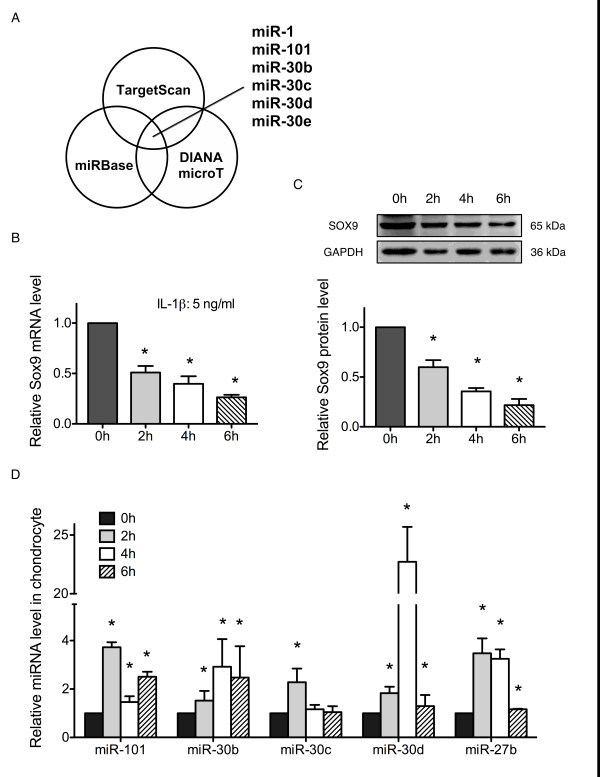
**microRNA prediction and its expression in IL-1β-treated chondrocytes**. (**A**) Six microRNAs were selected to target *Sox9 *using three classic prediction databases. Primary rat chondrocytes were treated with IL-1β (5 ng/ml) for 2, 4 and 6 h. *Sox9 *expression was analyzed by real-time PCR and western blot. (**B **and **C**) *Sox9 *expression was markedly decreased at the mRNA and protein levels with the increasing duration of IL-1β stimulation. The upper panels (**C**) are representative images of the western blot analysis. The lower panels are densitometric analyses performed with images of three independent experiments, respectively; *n *= 3,*P < 0.05 versus 0 h. (**D**) microRNAs expression in IL-1β-induced chondrocytes was analyzed by real-time PCR; *n *= 3,*P < 0.05 versus 0 h.

To investigate which miRNA might target *Sox9*, we first searched three miRNA prediction databases: TargetScan [25], miRbase [26] and DIANA-microT [27]. Six miRNAs (miR-1, miR-101, miR-30b, miR-30c, miR-30d, and miR-30e) were selected to potentially target *Sox9 *(Figure 1A). Second, we used IL-1β-treated rat chondrocytes (at different time points) as a screening platform to investigate which miRNAs were correlated with IL-1β and *Sox9 *during chondrocyte ECM degradation. A previous study has shown that IL-1β markedly down-regulates *Sox9 *expression in chondrocytes [7]. Similarly, our result showed a significant decrease in the *Sox9 *levels in rat chondrocytes after IL-1β stimulation at both the mRNA and protein levels (Figures 1B and 1C). Finally, these miRNAs were evaluated using real-time PCR to quantify the changes in their expression after IL-1β treatment at 2, 4, and 6 h, respectively. The expression of miR-101, miR-30b, miR-30c, miR-30d and miR-27b was detected (Figure 1D) but miR-1 and miR-30e expression was not (data not shown). miR-27b was used as a positive control as previously reported [19]. The increasing expression of miR-101, miR-30b, miR-30c, and miR-30d emerged at different time points after IL-1β treatment (Figure 1D). These results suggested a correlation between the increased expression levels of miR-101, miR-30b, miR-30c, and miR-30d, as well as the decreased *Sox9 *expression level. To determine whether the *in silico *analysis predicted the miRNA-targeting of *Sox9 *mRNA in a cellular environment, we performed luciferase assays and examined the changes in the *Sox9 *levels after miRNA transfection.

### Analysis and verification of miRNA target sites

**Figure 2:**
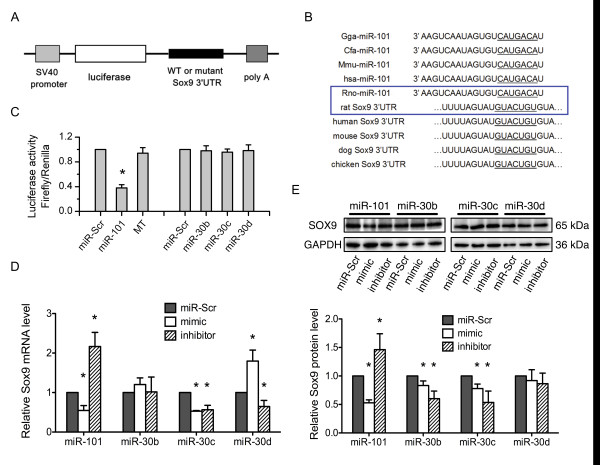
**Validation of the targeting effect of microRNAs on *Sox9***. (**A**) The diagram illustrates the construction of *Sox9 *3' UTR luciferase reporter. (**B**) The conserved sequences of miR-101 and the 3'UTRs of *Sox9 *in different species are compared. The underlined sequences indicate a sequence complementary in miR-101 to a specific binding site within the 3'UTR of *Sox9*. The sequences in the frame were used in our study. (**C**) Luciferase activity of the *Sox9 *3'UTR reporter was analyzed in HeLa cells. miRNAs were co-transfected with the wild-type *Sox9 *3' UTR or mutant vector. Scrambled 22 nt mimic (miR-Scr) was used as a negative control; *n *= 3,*P < 0.05 versus miR-Scr group. MT refers to mutant *Sox9 *3'UTR reporter. Primary rat chondrocytes were transfected with the miRNA mimic and miRNA inhibitor. miR-Scr was used as a negative control. *Sox9 *expression was analyzed by real-time PCR and western blot 24 h post miRNA transfection (**D **and **E**). Lower panels (**E**) show the densitometric analysis of *Sox9 *expression (upper panels), which is normalized by glyceraldehyde-3-phosphate dehydrogenase (GAPDH). The analysis was performed using images from three independent experiments; *n *= 3,*P < 0.05 versus miR-Scr group.

The miR-Report luciferase reporter was constructed to determine whether miRNAs could directly target the 3' UTR of *Sox9 *(Figure 2A). The reporter was co-transfected with miRNA mimics. A scrambled 22 nt mimic, miR-Scr, (with no homology to the mammal genome) was used as a control. Reporter activity was not affected by the scrambled mimic (miR-Scr). The miR-101 mimic significantly reduced the luciferase activity in the wild-type *Sox9 *3'UTR reporter but not in the mutant reporter (Figure 2C). Further analysis indicated that the *Sox9 *3'UTR contained the miR-101 target site, and these binding regions were conserved in different species (Figure 2B). However, luciferase activity was not reduced with the mimics of miR-30b, miR-30c, and miR-30d (Figure 2C). To verify *Sox9 *a target of miR-101 in primary chondrocytes, chondrocytes were transfected with the miRNA mimic and miRNA inhibitor. miR-Scr was used as a negative control. *Sox9 *expression was evaluated by real-time PCR and western blot analysis. miR-101 repressed the *Sox9 *expression, whereas the inhibition of miR-101 increased the *Sox9 *expression at both mRNA and protein levels in rat chondrocytes (Figures 2D and 2E). However, no negative regulatory effects on *Sox9 *expression were observed in miR-30b, miR-30c and miR-30d on (Figures 2D and 2E). Combined with the luciferase assay results, we proposed that only miR-101 directly targets *Sox9*, thereby negatively regulating *Sox9 *expression. Recently, miR-145 has been described to target *Sox9 *in chondrocytes [28,29]. By assessing the miR-145 expression in the presence of IL-1β, there was no significant increased miR-145 level observed, and no time- or concentration-dependent manner in our experimental system (Figure S1 in Additional file 1). Furthermore, we examined miR-101 and *Sox9 *expression level at different concentrations of IL-1β. We found that the increasing miR-101 expression had an IL-1β concentration-dependent effect on chondrocytes at 6 h and was correlated with *Sox9 *expression (Figure S2 in Additional file 1). These further validate the abovementioned finding that miR-101 directly targets *Sox9*. All subsequent work was then focused on miR-101.

### Effects of miR-101 on IL-1β-induced ECM degradation

To further analyze the effect of miR-101 on IL-1β-induced ECM degradation, the chondrocytes were transfected with miR-Scr, miR-101 mimic (mimic), or miR-101 inhibitor (inhibitor with a complementary sequence of miR-101). The cells were then treated with or without IL-1β 12 h post-miRNA transfection. The average miR-101 expression level post-miR-101 mimic transfection reached 300-fold that of the miR-Scr group. However, the average miR-101 level was 0.56-fold that of the miR-Scr group (Figure S3A in Additional file 1). The chondrocytes exhibited an elongated fibroblast-like morphology and decreased cell density in response to IL-1β treatment (Figure S4 in Additional file 1). Interestingly, the morphological changes and decreased cell density of the chondrocytes were likewise seen after miR-101 mimic transfection, regardless of whether they were treated with IL-1β or not (Figure S4 in Additional file 1). By contrast, chondrocytes maintained their spherical shape after transfection of the miR-101 inhibitor. The cell density was not significantly decreased, regardless of IL-1β treatment (Figure S4 in Additional file 1).

**Figure 3:**
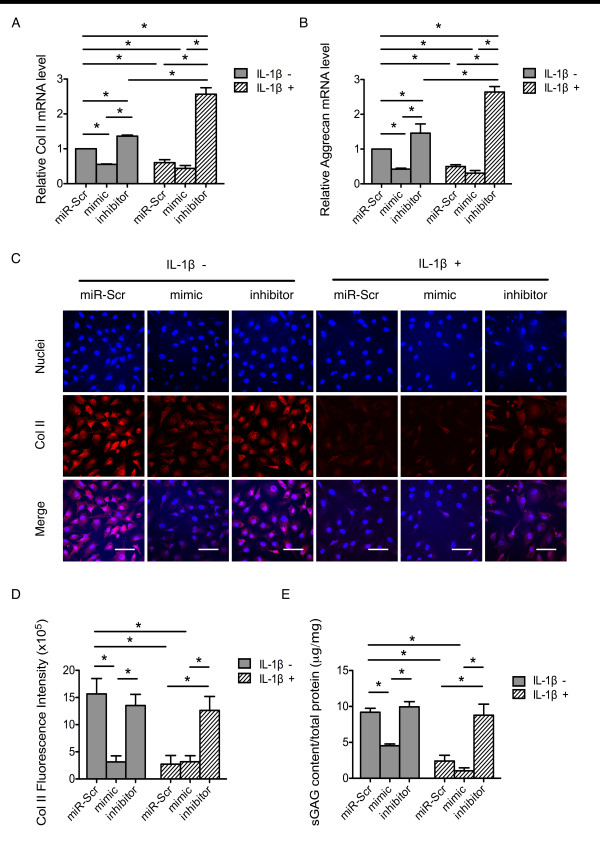
**Effects of miR-101 on IL-1β-induced chondrocyte extracellular matrix (ECM) degradation**. Primary rat chondrocytes were transfected with miR-Scr, miR-101 mimic, and miR-101 inhibitor, and then treated with or without IL-1β 12 h post-miRNA transfection. (**A **and **B**) The expression of *collagen type II *(**A**) and *aggrecan *(**B**) was evaluated by real-time PCR after transfection of miR-101 mimic or inhibitor with or without IL-1β treatment; *n *= 3, *P < 0.05. (**C**) The collagen type II expression of transfected chondrocytes was assessed by immunofluorescence staining method. Original magnification: × 60. Bars = 50 μm. (**D**) The fluorescence intensity of the images **(C**) was analyzed using Image-Pro Plus 6.0 software. Data are expressed as the average of at least five images; *n *= 3, *P < 0.05. (**E**) The sGAG content in the cell suspension was assessed by dimethylmethylene blue (DMMB) assay after transfection of miR-101 mimic or inhibitor with or without IL-1β treatment. Data were normalized by total protein content of the cell lysate in each group; *n *= 3, *P < 0.05.

*Collagen type II *and *aggrecan *are chondrocyte ECM genes that are down-regulated during chondrocyte ECM degradation [12,30,31]. In the present study, IL-1β inhibited the expression of *collagen type II *and *aggrecan *(Figure 3A and 3B, respectively). Similarly, the expression of *collagen type II *and *aggrecan *decreased after miR-101 transfection (Figure 3A and 3B, respectively). However, the expression of these two genes increased after miR-101 inhibitor transfection, regardless IL-1β treatment (Figure 3A and 3B, respectively). This result indicates that silencing miR-101 in chondrocytes can reverse the IL-1β-induced down-regulated expression of *collagen type II *and *aggrecan*. However, inhibiting miR-101 expression also increased *collagen type II *in the untreated chondrocytes (Figure 3A), indicating that the untreated chondrocytes have already expressed miR-101. Indeed, real-time PCR and northern blot analysis confirmed that the untreated chondrocytes expressed miR-101 (Figure S3B and S3C in Additional file 1). The level of miR-101 was lower than that of huh7 cells but higher than that of HeLa cells; both cell lines have been documented to have the basal miR-101 expression [32].

To assess the content changes of chondrocyte ECM, the secreted collagen type II content was evaluated by immunofluorescence staining. The DMMB assay was used to evaluate the concentration of sulfated-glycosaminoglycan (sGAG), a main form of aggrecan secreted by chondrocytes in cartilage. Similar to the abovementioned mRNA changes, the collagen type II and sGAG concentrations decreased after IL-1β simulation (Figures 3C, 3D and 3E). Overexpression of miR-101 decreased collagen type II and sGAG concentrations, regardless of IL-1β treatment (Figures 3C, 3D and 3E). However, silencing of miR-101 maintained collagen type II and sGAG content, which inhibited the effects of IL-1β (Figures 3C, 3D and 3E).

These results suggested that miR-101 mediate IL-1β-induced down-regulation of *collagen type II *and *aggrecan*, thereby affecting the changing concertrations of collagen type II and sGAG. Based on previous studies [7,9-11], *Sox9 *can directly promote the expression of *collagen type II *and *aggrecan*, whereas decreased *Sox9 *expression can down-regulate these two genes in the presence of IL-1β. Based on the combined results, mediation of these effects by miR-101 via *Sox9 *regulation should be investigated.

### miR-101 mediate IL-1β-induced down-regulation of *collagen type II *and *aggrecan*, probably by targeting *Sox9*

**Figure 4:**
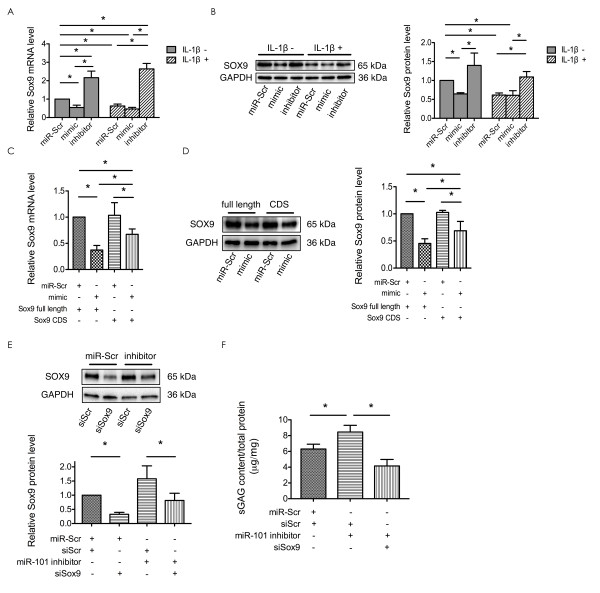
**miR-101 function during IL-1β-induced chondrocyte extracellular matrix (ECM) degradation probably occurs through regulation of *Sox9***. Primary rat chondrocytes were transfected with miR-Scr, miR-101 mimic, and miR-101 inhibitor, and then treated with or without IL-1β 12 h post-miRNA transfection in the same manner as mentioned above. (**A **and **B**) *Sox9 *expression was analyzed by real-time PCR (**A**) and western blot (**B**) 24 h post miRNA mimic, inhibitor, or miR-Scr transfection. The right panel (**B) **is the densitometric analysis of *Sox9 *expression (left panel), normalized by glyceraldehyde-3-phosphate dehydrogenase (GAPDH). The analysis was performed using images of three independent experiments, respectively; *n *= 3, *P < 0.05. (**C **and **D**) Primary rat chondrocytes were co-transfected with miR-Scr or miR-101 mimic together with *Sox9 *full-length vector (*Sox9 *expression vector containing both UTR and CDS regions) or *Sox9 *CDS vector (*Sox9 *expression vector containing only CDS region, no UTR). *Sox9 *expression was analyzed by real-time PCR (**C**) and western blot (**D**) 24 h post transfection; *n *= 3,*P < 0.05. (**E **and **F**) Primary rat chondrocytes were first transfected with miR-Scr or miR-101 inhibitor, and then siRNA against *Sox9 *(siSox9) or scrambled siRNA (siScr) were transfected. *Sox9 *expression was analyzed by western blot (**E**) 24 h post transfection. The lower panel (**E**) is the densitometric analysis of *Sox9 *expression (upper panel), normalized by GAPDH. The analysis was performed using images of three independent experiments, respectively; *n *= 3,*P < 0.05. **(F**) The sGAG content in the cell suspension was assessed by dimethylmethylene blue (DMMB) assay. Data were normalized by total protein content in the cell lysate in each group; *n *= 3,*P < 0.05.

We have shown that overexpression of miR-101 decreases the level of ECM gene expression as well as synthesis of collagen type II and aggrecan. Furthermore, silencing miR-101 inhibits these effects. Whether *Sox9 *participates in these processes remains unknown. Thus, *Sox9 *expression at both mRNA and protein levels was first assessed in the abovementioned treated chondrocytes. The results showed that IL-1β reduced *Sox9 *expression at both the mRNA and protein levels (Figure 4A and 4B). Furthermore, overexpression of miR-101 reduced *Sox9 *expression, whereas silencing of miR-101 increased *Sox9 *expression, whether the cells were treated with IL-1β or not (Figure 4A and 4B). Expression of *Sox9 *paralleled the expression of *collagen type II *and *aggrecan*. In addition, silencing miR-101 expression could reverse the down-regulation of *Sox9 *that was caused by IL-1β (Figure 4A and 4B). These results indicated that *Sox9 *participates in the miR-101 function during IL-1β-induced chondrocyte ECM degradation. To confirm if the effects of miR-101 were achieved by regulating *Sox9*, firstly, chondrocytes were co-transfected with miR-101 mimic or miR-Scr together with *Sox9 *vector with (*Sox9 *full length) or without (*Sox9 *CDS) the 3'UTR sequence. The expression of *Sox9 *was reduced in the chondrocytes that were co-transfected with miR-101 mimic and *Sox9 *full-length vector. The effect of miR-101 was partly reversed in the chondrocytes co-transfected with *Sox9 *CDS vector without 3'UTR for miR-101 binding (Figure 4C and 4D). Secondly, chondrocytes were first transfected with the miR-101 inhibitor and then transfected with siSox9, miR-Scr and siScr were used as negative controls. The expression of *Sox9 *was significantly reduced by siSox9, and the increased *Sox9 *level post miR-101 inhibitor transfection was reduced by co-transfection with siSox9 (Figure 4E). Moreover, an increase in sGAG content was observed after transfection with the miR-101 inhibitor alone; however, there was a remarkable decrease in sGAG content after co-transfection with miR-101 inhibitor and siSox9 (Figure 4F), thereby suggesting that *Sox9 *might be a functional mediator of the miR-101-mediated changes in sGAG concentration. Combined with the abovementioned results, the miR-101-mediate IL-1β-induced down-regulation of *collagen type II *and *aggrecan *was probably achieved by regulating its target gene *Sox9*.

## Discussion

The degradation of cartilage in OA is characterized by two phases: a degradative and a biosynthetic phase [2,33,34]. In the degradative phase, the enzymes, a disintegrin and metalloproteinase with thrombospondin motifs (ADAMTS) and matrix metalloproteinases (MMPs), produced by chondrocytes digest the ECM in the presence of inflammatory cytokines; matrix synthesis is likewise inhibited by inflammatory factors through the down-regulation of ECM genes. In the biosynthetic phase, the chondrocytes, which are the only cell types in cartilage, attempt to repair the damaged ECM. However, these cells cannot synthesize enough ECM because of the low level of ECM gene expression. Consequently, the erosion of the cartilage is accelerated. Evidence has revealed that miRNAs are associated with the expression of ECM degradation enzymes in the presence of IL-1β. Miyaki, *et al. *[17] reported that miR-140 could down-regulate *ADAMTS-5 *expression in IL-1β-induced OA chondrocytes. Tardif, *et al. *[18] and Akhtar, *et al. *[19] demonstrated that miR-27b inhibits the expression of *MMP-13 *in IL-1β-induced OA chondrocytes. However, to date, evidence of miRNAs participation during the down-regulation of ECM genes in the presence of IL-1β has not been well documented.

In the current study, we provide a new insight on the miRNAs that are involved in IL-1β-induced chondrocyte ECM degradation. We demonstrate that miR-101 mediate IL-1β-induced down-regulation of *Sox9*, and its known down-stream genes *collagen type II *and *aggrecan*; however, silencing miR-101 can reverse the IL-1β-induced down-regulation of these two genes as well as the degradation of the ECM proteins, collagen type II and sGAG. Thus, miR-101 may serve as a new target for preventing the IL-1β-induced chondrocyte ECM degradation.

The effect of miR-101 on the IL-1β-induced chondrocyte ECM degradation is probably achieved through *Sox9 *regulation. The following evidence support this hypothesis: First, the expression of miR-101 is negatively correlated with *Sox9 *expression, and the decreased *Sox9 *expression is due to the overexpression of miR-101 by targeting its 3'UTR. Second, *Sox9 *is a target of miR-101, and can directly promote ECM gene expression and ECM synthesis [9-11]. The decreased *Sox9 *expression can lead to the down-regulation of *collagen type II *and *aggrecan *in the presence of inflammatory cytokines [12]. Third, the increased content of sGAG caused by silencing miR-101 expression was significantly decreased by co-transfection with siSox9. However, miR-101 has no effects on the other aspects of *Sox9 *regulation, such as *p38*. It has been reported that *p38 *can regulate *Sox9 *[35], but miR-101 did not affect the *p38 *level (Figure S5C in Additional file 1), suggesting that the effect of miR-101 on *Sox9 *may be mainly through the direct targeting.

We also found that miR-101 could be induced by IL-1β, which is a direct effect demonstrated as a concentration-dependent effect on the endogenous miR-101 level (Figure S2A, S2B and S2C in Additional file 1). The regulation of IL-1β on miR-101 is at transcriptional level, because IL-1β can lead to an increased level of pre-miR-101 (Figure S3D in Additional file 1). However, miR-101 has no influence on the classic components of IL-1β pathway such as total *NF-kB *and the nuclear translocation of *nuclear factor (NF)-kB *(Figure S5D and S5E in Additional file 1).

Inhibiting miR-101 expression notably resulted in increased collagen type II synthesis in untreated chondrocytes. This phenomenon may indicate that chondrocytes have already expressed miR-101. Thus, we examined the basal levels of miR-101 in primary chondrocytes. As expected, we found that untreated chondrocytes had already expressed miR-101 (Figure S3B and S3C in Additional file 1).

miR-145 has been reported to target *Sox9 *in chondrocytes [28,29]. However, in the current study, we did not observe a negative correlation between the expression of miR-145 and *Sox9 *in the presence of IL-1β. The IL-1β treatment did not have any time- or concentration-dependent effects on miR-145 (Figure S1A and S1B in Additional file 1). Therefore, our work focused on miR-101.

However, it was noted that expression of *Sox9 *continued to decline with increasing duration of IL-1β treatment. The expression levels of miR-101 exhibited a decrease at 4 h and 6 h of IL-1β treatment compared to the levels at 2 h, although miR-101 expression remained higher than at 0 h. This finding may indicate that the reduced *Sox9 *level was not completely regulated by miR-101. Furthermore, miR-101 may have other targets in these processes. Further research is necessary to obtain additional information on miR-101 function.

miR-101 has been associated with cancer [24,36,37] and immune response [38]. Our findings provide evidence that miR-101 might participate in inflammation and cause chondrocyte ECM degradation.

## Conclusions

miR-101 is involved in IL-1β-induced down-regulation of *collagen type II *and *aggrecan*, and its inhibition can prevent IL-1β-induced chondrocyte ECM degradation. This miRNA probably function through its target gene *Sox9*.

## Abbreviations

ADAMTS: a disintegrin and metalloproteinase with thrombospondin motifs; ANOVA: analysis of variance; DMEM: Dulbecco's modified Eagle's medium; DMMB: dimethylmethylene blue; ECM: extracellular matrix; GAPDH: glyceraldehyde-3-phosphate dehydrogenase; IL: interleukin; miRNA: microRNA; MMP: matrix metalloproteinase; NF: nuclear factor; OA: osteoarthritis; PBS: phosphate-buffered saline; PCR: polymerase chain reaction; sGAG: sulfated-glycosaminoglycan; siRNA: small interfering RNA.

## Competing interests

The authors declare that they have no competing interests.

## Authors' contributions

LHD contributed to conception and design, acquisition of data, analysis and interpretation of data and drafting the manuscript. XZ and XQH were involved in data interpretation, statistical analysis and manuscript preparation. CYZ and YFA conceived the study, participated in its design and coordination, and helped to draft the manuscript. All authors contributed to revising the manuscript critically for important intellectual content, and have read and approved the manuscript for publication.

## Supplementary Material

Additional file 1 **Supplemental figures and figure legends**. This file contains 5 figures (Figure S1-S5) and their figure legends. Figure S1: Relative miR-145 expression levels at present of IL-1β in primary rat chondrocytes. Figure S2: miR-101 has an IL-1β concentration dependent effect in primary chondrocyte. Figure S3: miR-101 and the pri-miR-101 expression level. Figure S4: Morphological changes of the chondrocytes post miRNA transfection and IL-1β treatment. Figure S5: Effect of miR-101 transfection on the components of IL-1β signaling. Click here for file
