# Supplementary material for: Silencing of microRNA-101 prevents IL-1β-induced extracellular matrix degradation in chondrocytes
Source: Arthritis Res Ther. 2012 Dec 10;14(6):R268. doi: 10.1186/ar4114 (PMC3674628; doi:10.1186/ar4114)
Supplement: Additional file 1 — Supplemental figures and figure legends. This file contains 5 figures (Figure S1-S5) and their figure legends. Figure S1: Relative miR-145 expression levels at present of IL-1β in primary rat chondrocytes. Figure S2: miR-101 has an IL-1β concentration dependent effect in primary chondrocyte. Figure S3: miR-101 and the pri-miR-101 expression level. Figure S4: Morphological changes of the chondrocytes post miRNA transfection and IL-1β treatment. Figure S5: Effect of miR-101 transfection on the components of IL-1β signaling. [file ar4114-S1.PDF]

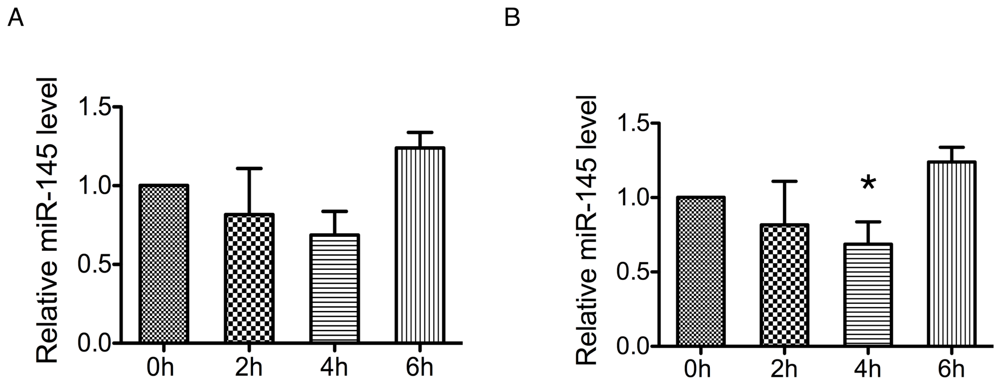

Figure S1 Relative miR-145 expression levels at present of IL-1β in primary rat chondrocytes

(A) Relative miR-145 expression was analyzed by real-time PCR after chondrocytes were treated with IL-1β (5 ng/ml) for 2, 4 and 6h. *n=3*,* *P* < 0.05 versus 0 h group.**(B)** Relative miR-145 expression was analyzed by real-time PCR after chondrocytes were treated at different concentration of IL-1β for 6h, respectively. *n=3*,* *P* < 0.05 versus 0 ng/ml group.

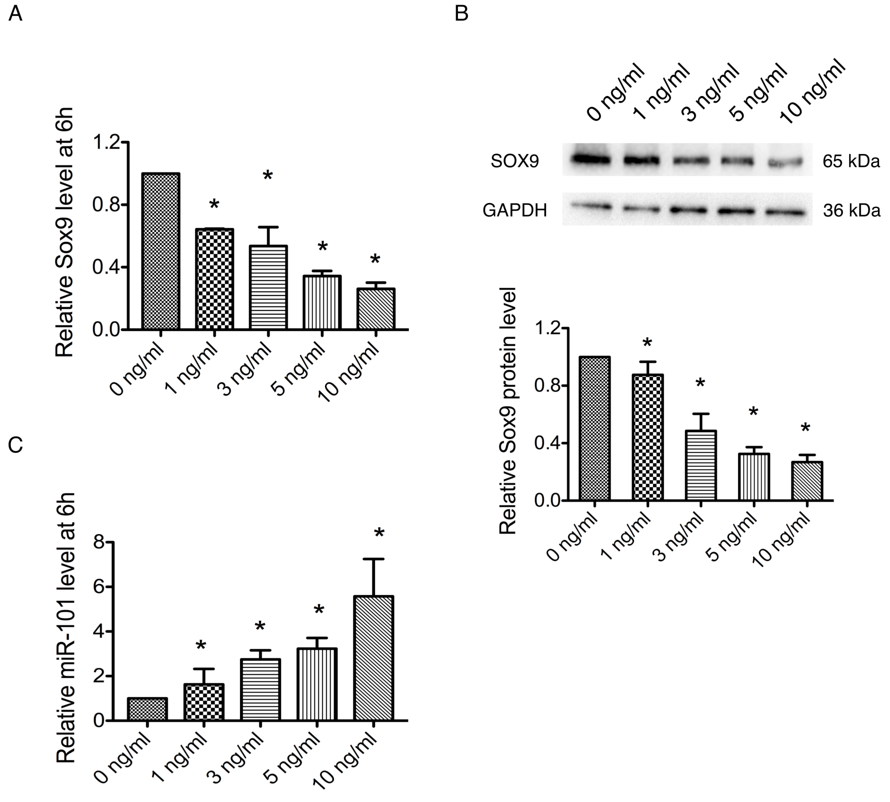

Figure S2 MiR-101 has an IL-1β concentration dependent effect in primary chondrocyte

Primary rat chondrocytes were treated at different concentration of IL-1β for 6h, respectively. **(A and B)** Sox9 expression was analyzed by real-time PCR **(A)** and Western blot **(B).** *n=3*,* *P* < 0.05 versus 0 ng/ml group. **(C)** MiR-101 expression at different concentration of IL-1β was accessed by real-time PCR. *n=3*,* *P* < 0.05 versus 0 ng/ml group.

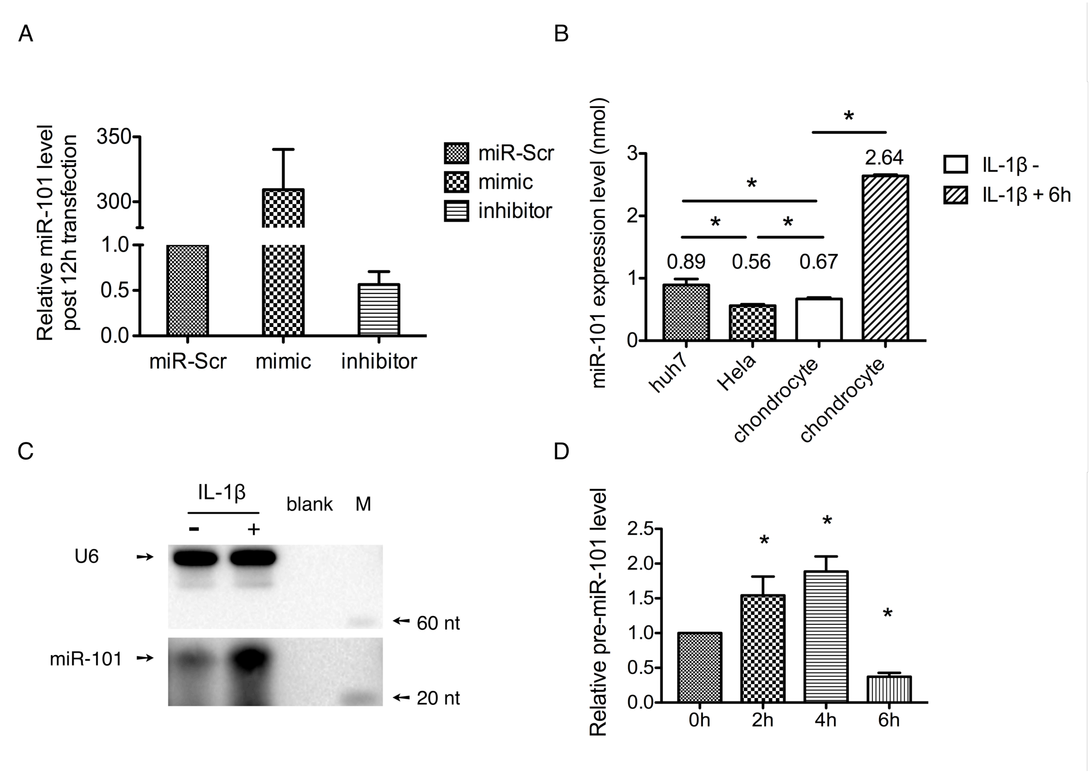

Figure S3 MiR-101 and the pri-miR-101 expression level

(A) Relative miR-101 expression was analyzed by real-time PCR post miR-Scr, miR-101 mimic and inhibitor transfection. **(B)** The miR-101 expression with or without IL-1β in chondrocytes was examined by real-time PCR using the absolute quantitative methods. We use huh7 and Hela cells as a positive control because these two cells have the basal miR-101 expression according to the database (www.microrna.org). *n=3*,* *P* < 0.05. **(C)** The miR-101 expression with or without IL-1β in chondrocytes was examined by northern blot. U6 was used as an internal control. M signs the molecular markers. **(D)** Relative pre-miR-101 expression was examined by real-time PCR after IL-1β treated for 2, 4 and 6h, respectively. *n=3*,* *P* < 0.05 versus 0 h group.

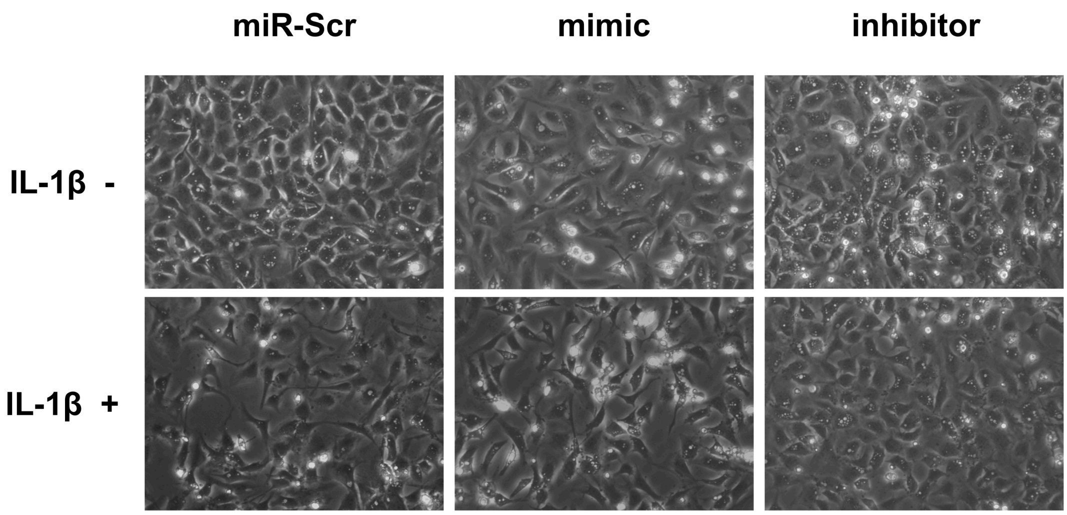

Figure S4 Morphological changes of the chondrocytes post miRNA transfection and IL-1β treatment

Primary rat chondrocytes were transfected with miR-Scr, miR-101 mimic, and miR-101 inhibitor, and then treated with or without IL-1β 12 h post-miRNA transfection. The morphological changes of the chondrocytes were examined under a microscope.

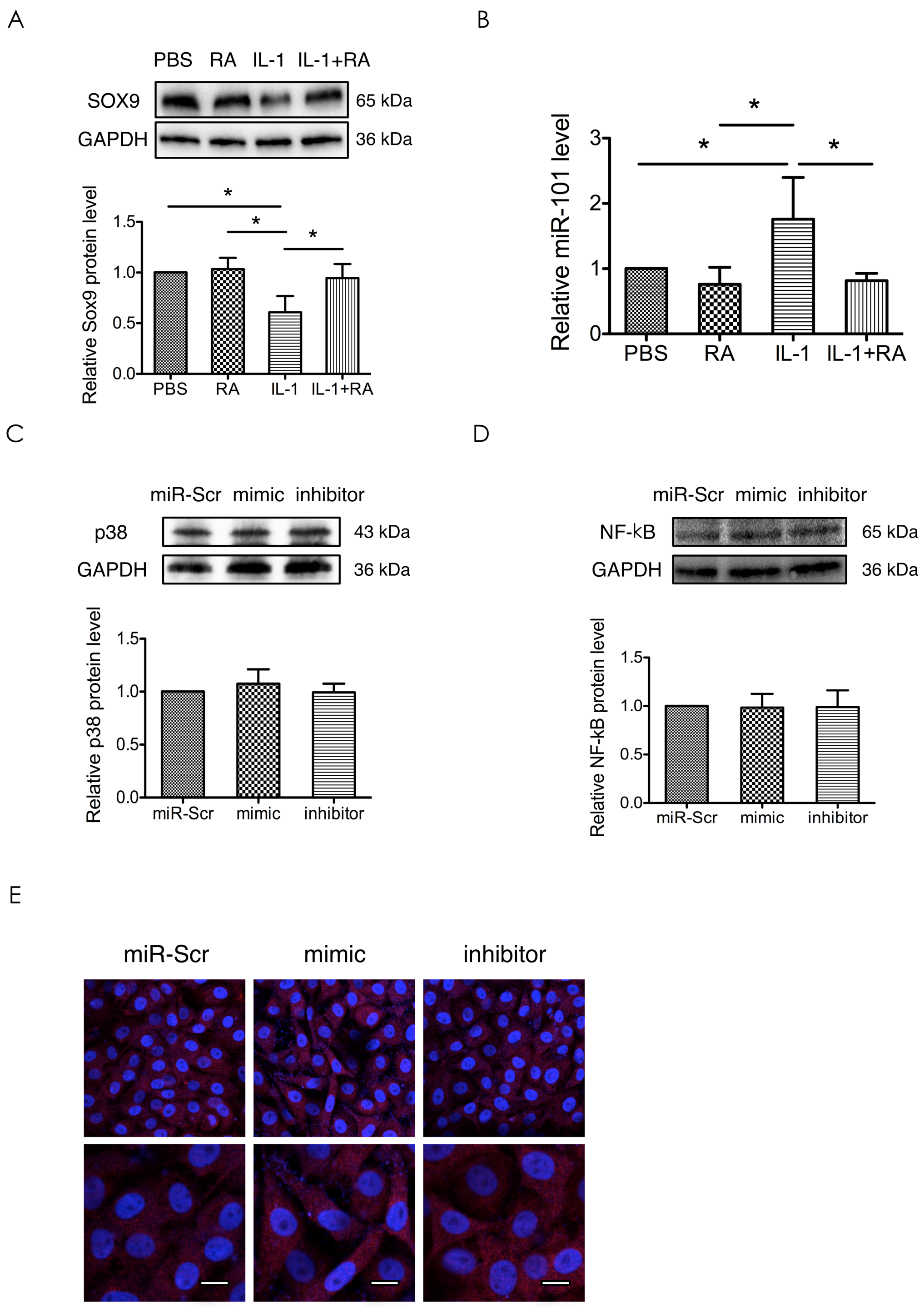

Figure S5 Effect of miR-101 transfection on the components of IL-1β signaling

(A and B) Primary chondrocytes were treated with PBS, IL-1β, IL-1Ra and IL-1β+IL-1Ra at 4h, respectively. Sox9 protein level was assessed by western blot **(A)** Upper panels of **(A)** are representative images of Western blot and lower panels are

densitometric analysis performed with images of three independent experiments, respectively. $n=3$,* $P < 0.05$. MiR-101 expression was analyzed by real-time PCR method **(B)**. **(C)** The protein level of p38 was assessed by western blot. Upper panels of **(C)** are representative images of Western blot and lower panels are densitometric analysis performed with images of three independent experiments, respectively. $n=3$,* $P < 0.05$. **(D)** P38 protein level was assessed by western blot. Upper panels of **(D)** are representative images of Western blot and lower panels are densitometric analysis performed with images of three independent experiments, respectively. $n=3$,* $P < 0.05$. **(E)** Nuclear translocation of NF-kB post-miR-101 transfection was examined by immunofluorescence staining method. Blue fluorescence labels the nuclear while the red fluorescence labels the NF-kB. The lower panels images are the enlarged images of the upper panels. Original magnification: x 60. Bars = 10 μm.
